# The Occurrence of a Commercial N^pro^ and E^rns^ Double Mutant BVDV-1 Live-Vaccine Strain in Newborn Calves

**DOI:** 10.3390/v10050274

**Published:** 2018-05-19

**Authors:** Kerstin Wernike, Anna Michelitsch, Andrea Aebischer, Uwe Schaarschmidt, Andrea Konrath, Hermann Nieper, Julia Sehl, Jens P. Teifke, Martin Beer

**Affiliations:** 1 Institute of Diagnostic Virology, Friedrich-Loeffler-Institut, Suedufer 10, 17493 Greifswald—Insel Riems, Germany; Anna.Michelitsch@fli.de (A.M.); Andrea.Aebischer@fli.de (A.A.); Martin.Beer@fli.de (M.B.); 2 Saxon State Laboratory of Health and Veterinary Affairs, Zschopauer Straße 87, 09111 Chemnitz, Germany; Uwe.Schaarschmidt@lua.sms.sachsen.de; 3 Saxon State Laboratory of Health and Veterinary Affairs, Bahnhofstraße 58-60, 04158 Leipzig, Germany; Andrea.Konrath@lua.sms.sachsen.de (A.K.); Hermann.Nieper@lua.sms.sachsen.de (H.N.); 4 Institute of Molecular Virology and Cell Biology, Friedrich-Loeffler-Institut, Suedufer 10, 17493 Greifswald—Insel Riems, Germany; Julia.Sehl@fli.de; 5 Department of Experimental Animal Facilities and Biorisk Management, Friedrich-Loeffler-Institut, Suedufer 10, 17493 Greifswald—Insel Riems, Germany; JensPeter.Teifke@fli.de

**Keywords:** bovine viral diarrhea virus, pregnancy, live vaccine, persistence, ear notch sampling

## Abstract

The major source for the spread of bovine viral diarrhea virus (BVDV) are in-utero infected, immunotolerant, persistently infected (PI) animals since they shed enormous amounts of viruses throughout their lives. During the sequence-based virus typing of diagnostic ear notch samples performed in the context of the obligatory German BVDV eradication program, the commercial N^pro^ and E^rns^ double mutant BVDV-1 live-vaccine strain KE-9 was detected in seven newborn calves; their mothers were immunized in the first trimester of gestation. Six calves either succumbed or were culled immediately, but the one remaining animal was closely monitored for six months. The viral RNA was detected in the skin sample taken in its first and fifth week of life, but the virus could not be isolated. Further skin biopsies that were taken at monthly intervals as well as every serum and urine sample, nasal, oral, and rectal swabs taken weekly tested BVDV negative. However, neutralizing titers against BVDV-1 remained at a consistently high level. To further control for virus shedding, a BVDV antibody and antigen negative calf was co-housed which remained negative throughout the study. The missing viremia, a lack of excretion of infectious virus and negative follow-up skin samples combined with consistently high antibody titers speak against the induction of the classical persistent infection by vaccination with recombinant KE-9 during gestation. We, therefore, suggest that the epidemiological impact of the RNA/antigen positivity for an extended period in the skin is very low. The detection of live-vaccine viruses in skin biopsies mainly represents a diagnostic issue in countries that implemented ear notch-based control programs; and KE9-specific RT-PCRs or sequence analysis can be used to identify these animals and avoid culling measures.

## 1. Introduction

Bovine viral diarrhea virus (BVDV), a pestivirus of the family *Flaviviridae* which is endemic in cattle populations worldwide, causes significant impact on animal welfare and major economic losses [1,2,3]. BVDV exists in two species, namely BVDV-1 and BVDV-2, and according to their growth in the cell culture, the virus isolates are classified into the two distinct biotypes cytopathic (cp) and non-cytopathic (ncp) [4,5]. Clinical signs of a BVDV infection range from unapparent infections or unspecific symptoms such as fever, diarrhea, pneumonia or hemorrhagic lesions to the inevitably fatal mucosal disease (MD). Fetal infection may, dependent on the phase of gestation, result in abortion, stillbirth, teratogenic effects, or, when the infection occurs during the first trimester, in the birth of immunotolerant, persistently infected, viremic calves [6,7]. These persistently infected (PI) animals are immunotolerant to the BVDV-strain they are infected with, making them unable to develop a specific immune response against this particular virus strain. As a result, PI animals shed enormous amounts of BVDV throughout their lives and they are the major source for the spread and perpetuation of the virus within individual cattle herds and for the transmission to previously not affected holdings [8,9,10]. For this reason, PI animals are the main target of bovine viral diarrhea (BVD) eradication programs, which have been implemented in several European countries [11,12,13,14]. 

In Germany, an obligatory nationwide control program has been in force since January 2011 and the defined basis rules are the obligatory testing of every newborn calf for the BVDV antigen or genome in the first 6 months of life. Since June 2016, testing has to be done in the first 4 weeks of life, all detected PI animals have to be immediately eliminated, and trade is only allowed with certified unsuspicious animals [14]. The majority of BVDV tests are carried out using ear biopsies taken during the tagging procedure which has to be done for every newborn calf in the European Union within its first seven days of life. Reinfections of cattle holdings are predominantly prevented by biosecurity measures, but in contrast to most countries with obligatory BVD control programs, voluntary vaccination is permitted in Germany and the vaccination of heifers is recommended to reduce the risk of infection of naïve pregnant animals. Inactivated preparations, as well as two different attenuated BVDV vaccines (Bovela^®^ (Boehringer Ingelheim Vetmedica GmbH, Ingelheim/Rhein, Germany); Vacoviron^®^ FS (Merial GmbH, Hallbergmoos, Germany)), are licensed in Germany [15].

Vacoviron^®^ includes the BVDV-1a vaccine virus Oregon C24V that was used since the 1960s in Europe [16,17]. Bovela^®^ received its marketing authorization in December 2014 and is based on the strains KE-9 (BVDV-1b) and NY-93 (BVDV-2a), where in both strains double individual genomic mutations were introduced in the N^pro^ protease and E^rns^ RNase (syn. E0) for attenuation [18]. The envelope protein E^rns^ and the nonstructural autoprotease N^pro^ are unique proteins which were only found in pestiviruses, but not in other members of the family *Flaviviridae* [19]. N^pro^ interferes with the host cellular alpha/beta interferon (IFN) response [20,21] and E^rns^, besides being an essential component of the pestiviral particle, possesses an intrinsic ribonuclease activity that can likewise inhibit the IFN response and assist in the development of persistent infections [21,22]. In the vaccine strains KE-9 and NY-93, two identical deletions were introduced, one in the N^pro^ gene prohibiting the protease from being expressed and the second one in the E^rns^ gene resulting in the abrogation of the ribonuclease function [18].

For the molecular tracing of virus transmission in the final phase of the German BVD eradication program, a sequence database of the circulating viruses was established by the German National BVDV Reference Laboratory (https://www.fli.de/de/home/).

In the context of the routine BVDV strain typing, viruses included in both live-vaccines were detected in ear notch samples of newborn calves whose mothers were immunized during pregnancy [23].

## 2. Materials and Methods 

Since 2008, BVDV-positive viral RNA or blood samples or ear notch samples soaked in either ELISA or PCR buffer have been submitted from veterinary diagnostic laboratories to the national reference laboratory for BVD/MD for molecular virus typing. The samples are routinely processed as described previously [23]. In cases of KE-9 or NY-93 detections, the double individual genomic deletions are confirmed by Sanger sequencing as described in Reference [23].

All but one calf in whose ear tissue samples vaccine virus was detected either succumbed or were culled immediately after the first positive BVDV test result according to the strict BVD regulation (regulation to protect cattle from bovine viral diarrhea virus infection; http://www.gesetze-im-internet.de/bvdvv/).

However, from one of the animals (calf number 6 in Table 1), a male Holstein-Frisian calf, subsequently named “Fiete”, a follow-up blood sample was taken after the initial BVDV positive ear notch sample. At the second sampling, a serum sample of the mother was taken as well. 

Twenty-four days after birth, Fiete was transported from the commercial cattle farm at which he was born to the Friedrich-Loeffler-Institut, Germany’s Federal Research Institute for Animal Health, and housed in a high-containment stable. Two days later, a BVDV antibody and antigen negative, male Holstein-Frisian calf aged 17 days and later on named “Volker” was co-housed to control for virus shedding of Fiete.

From Fiete’s 34th day of life onwards, blood and urine samples and nasal, oral, and rectal swabs were taken weekly. Swabs were collected in 2 mL Minimum Essential Medium (MEM). In addition, skin biopsies were taken at monthly intervals. Both calves were euthanized after 6 months and the following comprehensive panel of tissue samples was taken at necropsy: serum, blood, synovia; parenchyma: muscle, liver, kidney, heart, lung, pancreas, thymus, suprarenal gland; neural tissue: cerebrum, spinal cord, cerebellum; reticuloendothelial system: tonsil, spleen, bone marrow, mesenteric, mandibular, retropharyngeal and tracheobronchial lymph nodes; digestive tract: soft palate, salivary gland, esophagus, rumen, reticulum, omasum, abomasum, duodenum, jejunum, ileum including Peyer’s patches, caecum including ileocecal valve, colon, rectum; genitals: testis, vesicular, bulbourethral and prostate glands; skin/epithelia: ear, eyelid, muzzle, teat, nasal mucosa, trachea, urinary bladder. 

Leucocytes were isolated from anti-coagulated whole blood samples by centrifugation (1000 rpm, 5 min, 4 °C) after the specific lysis of erythrocytes by ammonium chloride (8.29 g/L lysis buffer). RNA was extracted from sera, leucocytes, urine, and swabs using the QIAamp Viral RNA Mini Kit (Qiagen GmbH, Hilden, Germany), from skin biopsies by the RNeasy Mini Kit (Qiagen, Hilden, Germany) and from further tissue samples using the King Fisher 96 Flex (Thermo Scientific, Braunschweig, Germany) in combination with the MagAttract Virus Mini M48 Kit (Qiagen, Hilden, Germany) according to the manufacturer’s instructions. The obtained RNA was subsequently tested by a panpesti real-time PCR [23]. The skin and serum samples were additionally tested by a commercial antigen ELISA (IDEXX BVDV Ag/Serum Plus Test, IDEXX, Liebefeld, Switzerland). 

For virus isolation, tissue samples were homogenized in 1 mL of MEM and inoculated onto BVDV antibody and virus-free bovine KOP-R cells (L0244, Collection of Cell Lines in Veterinary Medicine, Insel Riems, Germany).

Furthermore, the sera were analyzed by microneutralization tests against the BVDV-1b strain “Paplitz” and the BVDV-2a strain “CS8644” as described in the German official collection of test methods for bovine viral diarrhea [24] and by commercially available NS3- or E^rns^-based antibody ELISAs (ID Screen BVDV p80 Ab competition (ID.vet, Grabels, France), BVDV p80AB (IDEXX, Liebefeld, Switzerland), and Monoscreen AB ELISA BVDV (E0)/blocking (Bio-X Diagnostics, Rochefort, Belgium)). 

The initial ear notch samples of every calf and the follow-up blood sample of calf number 6 and its mother were taken by the responsible farm veterinarians in the context of the German mandatory BVD control program and as prescribed in the BVD regulation (Verordnung zum Schutz der Rinder vor einer Infektion mit dem Bovinen Virusdiarrhoe-Virus; http://www.gesetze-im-internet.de/bvdvv/). The protocol of the subsequent follow-up experiment has been reviewed by the responsible state ethics commission and was approved by the competent authority (State Office for Agriculture, Food Safety and Fisheries of Mecklenburg-Vorpommern, Rostock, Germany, permission number LALLF M-V/TSD/7221.3-2-016/17, 19 June 2017). Blood samples of calves whose ear notch samples tested BVDV negative and which received colostrum of their vaccinated mothers were taken by the responsible farm veterinarians in the context of the health monitoring program of the respective farms.

## 3. Results

### 3.1. KE-9 Detection in Ear Tissue Samples of Newborn Calves 

During the sequence-based virus typing of diagnostic ear notch samples, the recombinant type 1 virus (strain KE-9) included in the vaccine Bovela^®^ was detected in a total of seven calves of 1243 tested samples (see Table 1). Interestingly, the type 2 virus (strain NY-93) which is also part of the vaccine preparation has not been found in any of the ear tissue samples of newborn calves.

In each case of KE-9 detection, the double individual genomic deletions (N^pro^ protease codons 5 to 168, and E^rns^ RNase codon 349 [18]) introduced into the recombinant vaccine strain were confirmed by Sanger sequencing of ear tissue samples. In 4 cases, either RNA or ear tissue samples soaked in a PCR lysis buffer were submitted, consequently, virus isolation could not be attempted. From the tissue samples of the remaining 3 animals, the virus could not be isolated, although three blind passages were made. 

The mothers of the live-vaccine virus positive calves were immunized in the first trimester between day 21 and 66 of gestation (see Table 1), one dam was vaccinated twice during pregnancy (days 64 and 191). Six of the calves either succumbed or were culled immediately after the first positive BVDV test result according to the strict BVD regulation (regulation to protect cattle from bovine viral diarrhea virus infection; http://www.gesetze-im-internet.de/bvdvv/).

### 3.2. Long-Term Follow up of a Calf Whose Mother Was Vaccinated during Pregnancy

**Figure 1:**
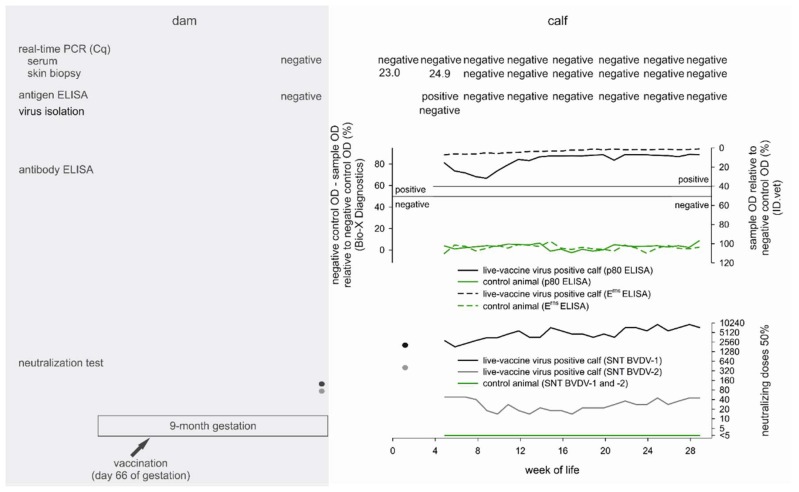
The virological and serological test results of a calf whose ear tissue sample tested positive for a BVDV live-vaccine virus. The gestation period and the test results of the mother are shaded in gray and the results of a co-housed BVDV-negative calf are shown in green. The cutoff values of the antibody ELISAs are marked by solid lines (Bio-X Diagnostics <50% negative and ≥50% positive; ID.vet ≥50% negative, >40% and <50% doubtful, and ≤40% positive). The black dots and lines in the lower figure panel represent neutralizing titers measured against BVDV-1 and gray dots and lines show neutralizing anti-BVDV-2 titers.

From Fiete, the remaining calf, a follow-up blood sample was taken after the initial BVDV positive ear notch sample (quantification cycle value [Cq]: 23.0). The follow-up blood sample tested negative for pestiviral RNA. Due to the disagreeing test results of the calf’s samples and the application of live-vaccines in the cattle herd, the samples were submitted for virus typing resulting in the confirmation of the presence of KE-9 vaccine virus RNA. Serum samples taken from Fiete’s mother tested negative both by real-time RT-PCR and antigen-ELISA, but neutralizing titers of 1/135 (BVDV-1) and 1/95 (BVDV-2) could be detected (Figure 1). 

Fiete appeared slightly retarded in physical growth compared to Volker, the in-contact calf. At necropsy, no gross lesions were detected.

Viral RNA could be detected in Fiete’s initial ear notch sample and in the skin biopsy taken on his 34th day of life (Cq value 24.9, see Figure 1). The antigen-ELISA, likewise, scored positive when testing the skin sample from day 34 (corrected optical density (OD) of 0.352, test cut-off >0.3), while virus could not be isolated out of this sample and the serum sample taken at the same day tested negative by RT-qPCR. Further skin biopsies and serum samples, as well as every swab and urine sample, tested BVDV negative. With the exception of the retropharyngeal lymph node (Cq value 37.2), every sample taken at necropsy tested negative by RT-qPCR. 

The BVDV-antibody levels measured by an NS3-based ELISA (ID.vet) dropped consistently from week 4 to week 9, but subsequently increased again and remained mostly constant from week 12 to the end of the study (see Figure 1). The results were confirmed by a second NS3-based antibody-ELISA (IDEXX). However, in an E^rns^-based antibody-ELISA (Bio-X Diagnostics), consistently high antibody levels were detected (see Figure 1). By microneutralization tests, titers of 1/1812 against BVDV-1 and 1/321 against BVDV-2 were determined from the first blood sample that was taken on Fiete’s 9th day of life. The neutralizing titer against BVDV-2 declined to 1/14 (week 10) and remained between 1/14 and 1/45 until the end of the study. The neutralizing titers measured against BVDV-1 remained at a consistently high level throughout the study (1/1812 to 1/9148, Figure 1). For comparison and to confirm that antibody levels against the species 1 and species 2 viruses included in the vaccine usually decline similarly, 10 calves whose ear notch samples tested BVDV negative and which received colostrum of their Bovela^®^ vaccinated mothers were blood sampled once between their 8th and 12th week of life and tested by neutralization assays. The neutralizing titers ranged between <1/5 and 1/11 (BVDV-1) and <1/5 and 1/28 (BVDV-2), respectively.

Samples taken from Volker scored consistently negative in each applied antibody (see Figure 1), antigen, or genome test.

## 4. Discussion

As previously demonstrated, some live BVDV vaccines including recombinant viruses which lack the function of only single viral proteins are able to pass the placental barrier and replicate in the fetus [25,26]. For the recombinant double mutants with a deletion of the N^pro^-protein and a mutation within the E^rns^, no vertical transmission was reported initially [26].

However, the recent first detection of the N^pro^ and E^rns^ double mutant live-vaccine virus in ear tissue samples [23] prompted the question of whether the immunization of pregnant dams with these vaccine strains could, in rare cases, induce PI animals, although no persistent infection attributable to the vaccination could be identified during several overdose studies [18]. Both proteins, N^pro^ and E^rns^, are non-redundant IFN antagonists and, thereby, are crucial for the establishment and maintenance of persistent pestiviral infections [21,26]. N^pro^ prevents IFN induction in BVDV-infected host cells, while E^rns^ prevents IFN induction in uninfected cells by targeting major pathogen-associated molecular patterns (PAMPs). Thus, BVDV maintains self-tolerance by efficiently avoiding the induction of IFN [21]. Nevertheless, immunization of pregnant cattle with the strain KE-9, deficient in functional E^rns^ and N^pro^ enzymes, during the well-defined timeframe for the induction of PI animals (between days 40 and 125 of gestation [27]) occasionally leads to the persistence of the vaccine virus in the fetus until birth (Table 1). However, no virus could be isolated from the skin samples of the newborns. Moreover, viral RNA was not detectable in the blood samples taken simultaneously with the PCR-positive ear tissue samples from the calf that was monitored for 6 months, which is in line with previous observations of missing viremia in calves in whose ear notch samples, the vaccine virus was found [23] and which argues against a classical persistent infection. Furthermore, the long-term monitored calf did not shed the KE-9 virus in any excretions or secretions and the in-contact animal did not get infected. In addition, the skin became PCR-negative after a few weeks, further indicating that the vaccine virus did not induce a classical persistent infection, but a kind of prolonged transient infection for several months with a presence restricted mainly to the skin. Therefore, the epidemiological impact of the detection of live-vaccine viruses in ear notch samples is very low, and most likely only represents a diagnostic issue in countries that implemented ear notch based control programs. Nevertheless, vaccination with the recombinant vaccine should be avoided during the critical period of pregnancy to prevent diagnostic issues when ear notch based BVDV control programs are in place.

Interestingly, only the type 1 virus included in the Bovela^®^ vaccine has been detected in newborn calves so far. The reasons for that observation remain unknown, but the type 2 virus could potentially induce abortion rather than long-term infection, especially since the parental virus of the double mutant is highly pathogenic and, although highly attenuated in the adult host, the mutant virus showed an enhanced capacity to induce fetal death after intrauterine infection [26].

Another key characteristic of persistent BVDV infections, besides the presence of the virus in high titers on successive occasions, is the lack of specific antibodies against the persisting virus [28,29]. Considering the vaccination of the dams and the transfer of maternally derived antibodies via the colostrum, the immune response of vaccine virus positive calves can only be assessed after the decline of the colostrum derived anti-BVDV antibodies whose mean half-life is about 23 days [30]. Thus, the initial drop of the BVDV-2 antibodies in sera collected from Fiete can be best explained by the reduction of maternally derived antibodies, while the following low-level persistence is the consequence of serological cross-reactivity that occurs between both BVDV species [31]. In contrast to the antibodies against BVDV-2, neutralizing titers against BVDV-1 remained at a consistent very high level in comparison to the control calves group from vaccinated cows, indicating that they were produced by the calf itself either in utero or after birth against KE-9. Hence, this constant level of neutralizing antibodies speaks against a persistent infection of the animal. However, like the RNA detection in ear notch samples, the antibody induction in calves by vaccination of their mothers could also interfere with the diagnostics in the context of BVDV control programs. When spot testing, that is the serological analysis of young stock (calves > 6 months of age), is applied to monitor for the BVDV status of cattle herds in countries with well-advanced control programs, antibody positive calves could mistakenly hint to new introductions of field viruses into the respective farms.

In summary, the missing viremia, a lack of excretion of infectious virus and negative follow-up skin samples combined with consistently high antibody titers demonstrate that the vaccination of dams with the recombinant KE-9 vaccine strain during gestation does not induce persistent infection, but a prolonged transient infection or the presence of the vaccine virus RNA and antigen for an extended period only in the skin of the newborn. It can be hypothesized that the skin is an immune-privileged compartment [32] which allows the long-term replication for months, despite the normally massive activation of an IFN response by the deletion of N^pro^ and the mutation of the E^rns^ protein in the recombinant BVDV vaccine strain KE-9. The mechanisms allowing for the efficient replication in the skin in these cases are yet unknown and should be further studied. In addition, it is also not known, whether the virus replicates in these cases within further immune-privileged organs in the fetus which should be studied by the experimental inoculation of pregnant cows in the future.

Nevertheless, the overall impact of the observed unexpected phenomenon of the long-term vaccine virus RNA/antigen positivity is very low, especially since only a very small number of animals is affected, and the detection of the live-vaccine viruses in skin samples mainly represents a diagnostic issue which can also be overcome by the re-testing of positive samples from calves of vaccinated mothers using vaccine-specific RT-PCRs. Furthermore, sequence analysis to differentiate between vaccine viruses and a potential re-introduction of a field-virus is recommended when BVDV is detected in ear notch samples of newborn animals in hitherto unsuspicious herds in which live vaccines have been applied.

## Figures and Tables

**Table 1:** Calves in whose ear notch samples live-vaccine viruses were detected after immunization of their mothers during pregnancy. The day of gestation when vaccinated was calculated from the vaccination date and insemination date (bold), or, if the conception date was not known, from the day of calving assuming a gestation period of 280 days (italic).

No. Calf	Day of Pregnancy When Vaccinated	Cq in Ear Notch Sample	Virus Isolation	Remarks
1	**64**	26.2	negative	culled, [23]
2	unknown	24.1	n.d. ^1^	succumbed after birth, [23]
3	*50*	24.5	n.d. ^1^	dead on its 14th day of life, [23]
4	*21*	26.1	n.d. ^1^	slaughtered
5	*38*	22.4	n.d. ^1^	slaughtered
6	**66**	23.0	negative	“Fiete”; further observed under experimental conditions
7	*64* and *191*	25.6	negative	slaughtered

^1^ n.d.—not done, Cq—quantification cycle value.
